# Utilizing electronic medical records alert to improve documentation of neonatal acute kidney injury

**DOI:** 10.1007/s00467-024-06352-2

**Published:** 2024-03-22

**Authors:** Arwa Nada, Amy Bagwell

**Affiliations:** 1 Department of Pediatrics, Division of Pediatric Nephrology, The University of Tennessee Health Science Center (UTHSC), 50 N Dunlap St., Memphis, TN 38105 USA; 2 Le Bonheur Children’s Hospital, Memphis, TN USA; 3 Department of Information Technology, Methodist Le Bonheur Health System, Memphis, TN USA

**Keywords:** Neonatal, AKI, Electronic medical records, Alerts

## Abstract

**Background:**

Neonatal acute kidney injury (AKI) is a common yet underdiagnosed condition in neonates with significant implications for long-term kidney health. Lack of timely recognition and documentation of AKI contributes to missed opportunities for nephrology consultation and follow-up, potentially leading to adverse outcomes.

**Methods:**

We conducted a quality improvement (QI) project to address this by incorporating an automated real-time electronic medical record (EMR)-AKI alert system in the Neonatal Intensive Care Unit (NICU) at Le Bonheur Children’s Hospital. Our primary objective was to improve documentation of neonatal AKI (defined as serum creatinine (SCr) > 1.5 mg/dL) by 25% compared to baseline levels. The secondary goal was to increase nephrology consultations and referrals to the neonatal nephrology clinic. We designed an EMR-AKI alert system to trigger for neonates with SCr > 1.5 mg/dL, automatically adding AKI diagnosis to the problem list. This prompted physicians to consult nephrology, refer neonates to the nephrology clinic, and consider medication adjustments.

**Results:**

Our results demonstrated a significant improvement in AKI documentation after implementing the EMR-AKI alert, reaching 100% compared with 7% at baseline (*p* < 0.001) for neonates with SCr > 1.5 mg/dL. Although the increase in nephrology consultations was not statistically significant (*p* = 0.5), there was a significant increase in referrals to neonatal nephrology clinics (*p* = 0.005).

**Conclusions:**

Integration of an EMR alert system with automated documentation offers an efficient and economical solution for improving neonatal AKI diagnosis and documentation. This approach enhances healthcare provider engagement, streamlines workflows, and supports QI. Widespread adoption of similar approaches can lead to improved patient outcomes and documentation accuracy in neonatal AKI care.

**Graphical Abstract:**

**Figure:**
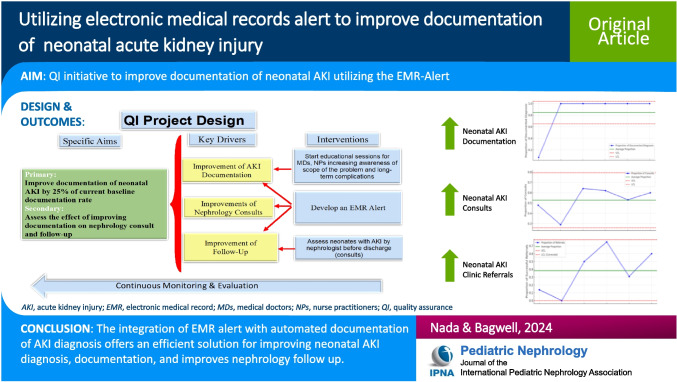
A higher resolution version of the Graphical abstract is available as Supplementary information

**Supplementary Information:**

The online version contains supplementary material available at 10.1007/s00467-024-06352-2.

## Introduction

Neonatal acute kidney injury (AKI), while it occurs in approximately 30% of neonates [1], is vastly underdiagnosed and under-documented in discharge charts [2, 3]. While the wider use of the modified neonatal Kidney Disease Improving Global Outcomes (KDIGO) AKI criteria was associated with improvement in recognition, the documentation of this common neonatal condition can still go unrecognized by the primary team with subsequent loss of nephrology consult and follow-up [2, 3]. Documentation of the diagnosis is equally important for clinicians and families to take the necessary measures of follow-up for these neonates, as they are at risk of developing long-term renal complications like hypertension, proteinuria, and chronic kidney disease (CKD). Additionally, missing the diagnosis from charts can underestimate the incidence of neonatal AKI, especially in studies relying on databases, like the Pediatric Health Information System (PHIS) database. This under-recognition and under-documentation is attributed to lack of awareness, variable standards of care, physicians’ work overload, absence of unified agreed-upon criteria of neonatal AKI across different institutes or even different providers in the same unit, lack of effective clinical decision support (CDS) tools in the electronic medical record (EMR) that help in diagnosis within the normal clinical workflow, and the reliance on human factors to document the diagnosis of AKI in discharge records which have always fallen short of perfect leading to documentation workarounds or missing critical patient information.

The use of CDS tools embedded within EMR in routine clinical care presents an important yet largely unfulfilled opportunity. Many hospitals are attempting to increase the efficiency of their operations and patient management by adopting clinical decision-support packages that enable the use of EMR data. EMR, which stores all medical information such as history, examination, laboratory tests, medications, procedures, and billing data, has been the most reliable medical data in the healthcare system [4]. Studies in adults have successfully demonstrated the effect of implementing diagnostic algorithms within EMR in identifying different medical condition risks and alerting physicians or pharmacists to take early actions as part of safety or quality improvement (QI) initiatives [5–7]. Two successful examples used in pediatric hospitals are the Nephrotoxic Injury Negated by Just in Time Action (NINJA) [8] and Baby NINJA [9].

EMR alerts have been increasingly utilized in recent years to improve documentation and recognition of various diseases and medical conditions. An EMR alert is a computer-based system integrated into the EMR of patients. It utilizes advanced algorithms and machine-learning techniques to analyze patient data and generate alerts for healthcare providers when a potential diagnosis is suspected. In AKI, the EMR alert system has the potential to analyze several factors, such as changes in serum creatinine (SCr) levels, urine output (UOP), medications, and comorbidities, to generate alerts. The utilization of AKI-EMR alerts with more advanced built-in automated documentation has several advantages. This can help healthcare providers recognize AKI in a timely manner, which is crucial for initiating appropriate management and preventing associated complications. Additionally, EMR alerts can improve documentation of AKI diagnosis in medical records, which can aid in accurate billing, data collection, and quality improvement initiatives and can decrease human error and missing important data documentation.

The primary goal of this QI project was to improve the diagnosis and documentation of neonatal AKI based on a SCr level of > 1.5 mg/dL by incorporating an automated real time EMR-AKI alert system. The second aim was to assess the effect of alerts on the number of nephrology consultations and referrals to the neonatal nephrology clinic.

## Methods

### Scope of the problem

The scope of the problem is under recognition and documentation of neonatal AKI in the problem list and discharge summary, with subsequent lack of nephrology involvement during admission and after discharge.

### QI design/objective/aim

**Fig. 1:**
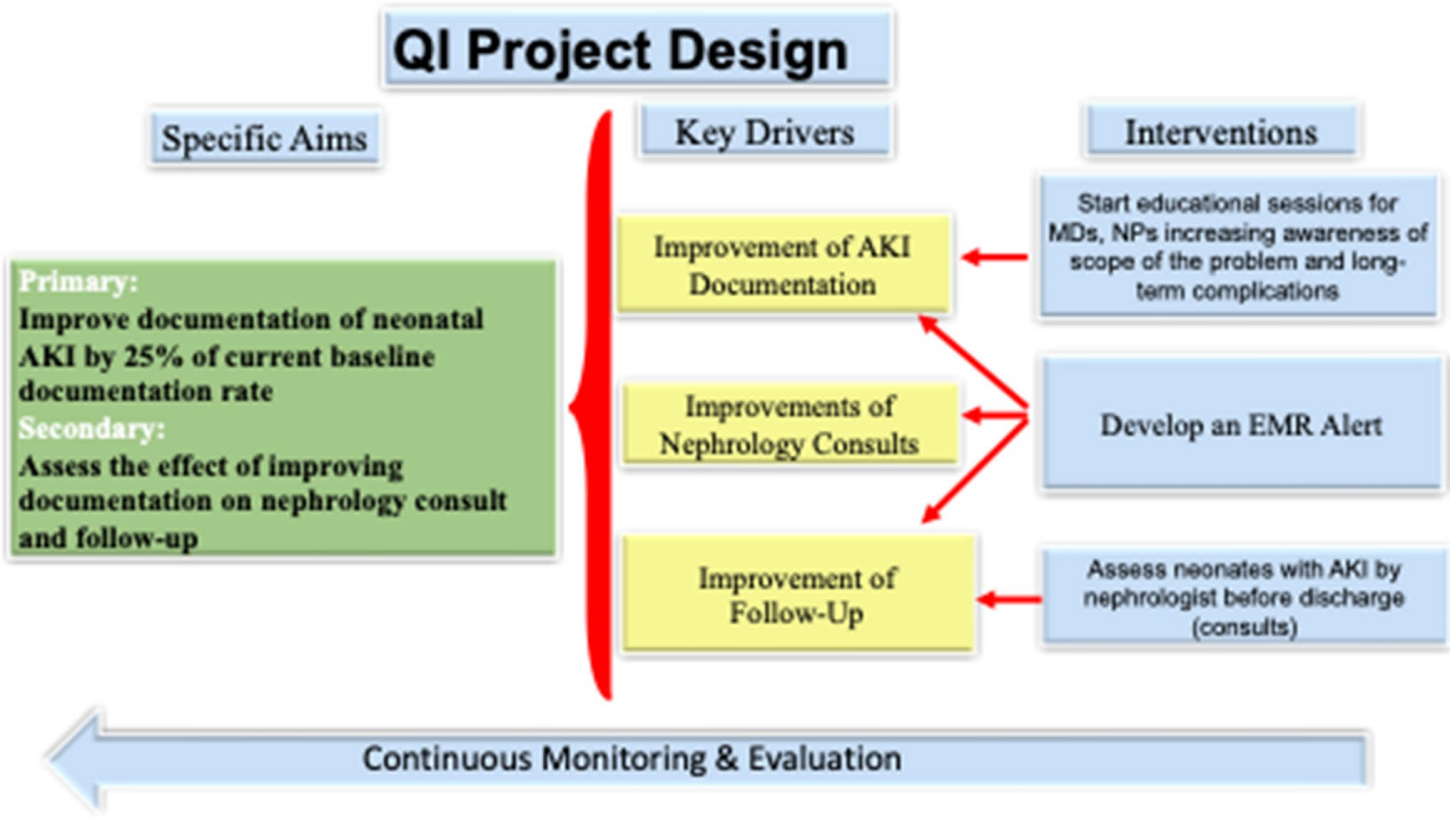
Quality improvement project design. *NPs*, nurse practitioners; *MDs*, medical doctors; *EMR*, electronic medical records; *AKI*, acute kidney injury

In July 2019, we initiated a QI project to enhance the diagnosis and documentation of neonatal AKI in the Neonatal Intensive Care Unit (NICU) at Le Bonheur Children’s Hospital in Memphis, TN, USA. Figure 1 outlines the key drivers and interventions used in the project. The Institutional Review Board (IRB) of the University of Tennessee Health Science Center approved this project. Obtaining consent was waived. The proposed intervention was creating an EMR Alert to automatically add the diagnosis of AKI to the problem list and serve as the pivot to facilitate nephrology consults and referrals to newborn-renal clinic.

This QI project followed the concept of a stepwise approach. This concept in QI projects in healthcare is well established and recognized as an effective strategy for implementing and managing change. This approach is epitomized by the Plan-Do-Study-Act (PDSA) model, which offers a structured framework for iterative testing and refinement of changes. This model includes the following steps: Plan: Develop the initiative to outline the objectives and plan the actions. Do: Implement the plan, where the planned actions are executed. Study: Check the results, where the outcomes of the actions are assessed and analyzed. Act: Make further improvements, where adjustments are made based on the findings from the “Study” phase [10].

### Aims

The primary aim was to improve documentation of neonatal AKI in newborns with SCr > 1.5 mg/dL by 25% of the current baseline documentation over first 12 months. The secondary goal was to improve nephrology consultation and referral to the neonatal nephrology clinic.

### Project design/intervention

**Fig. 2:**
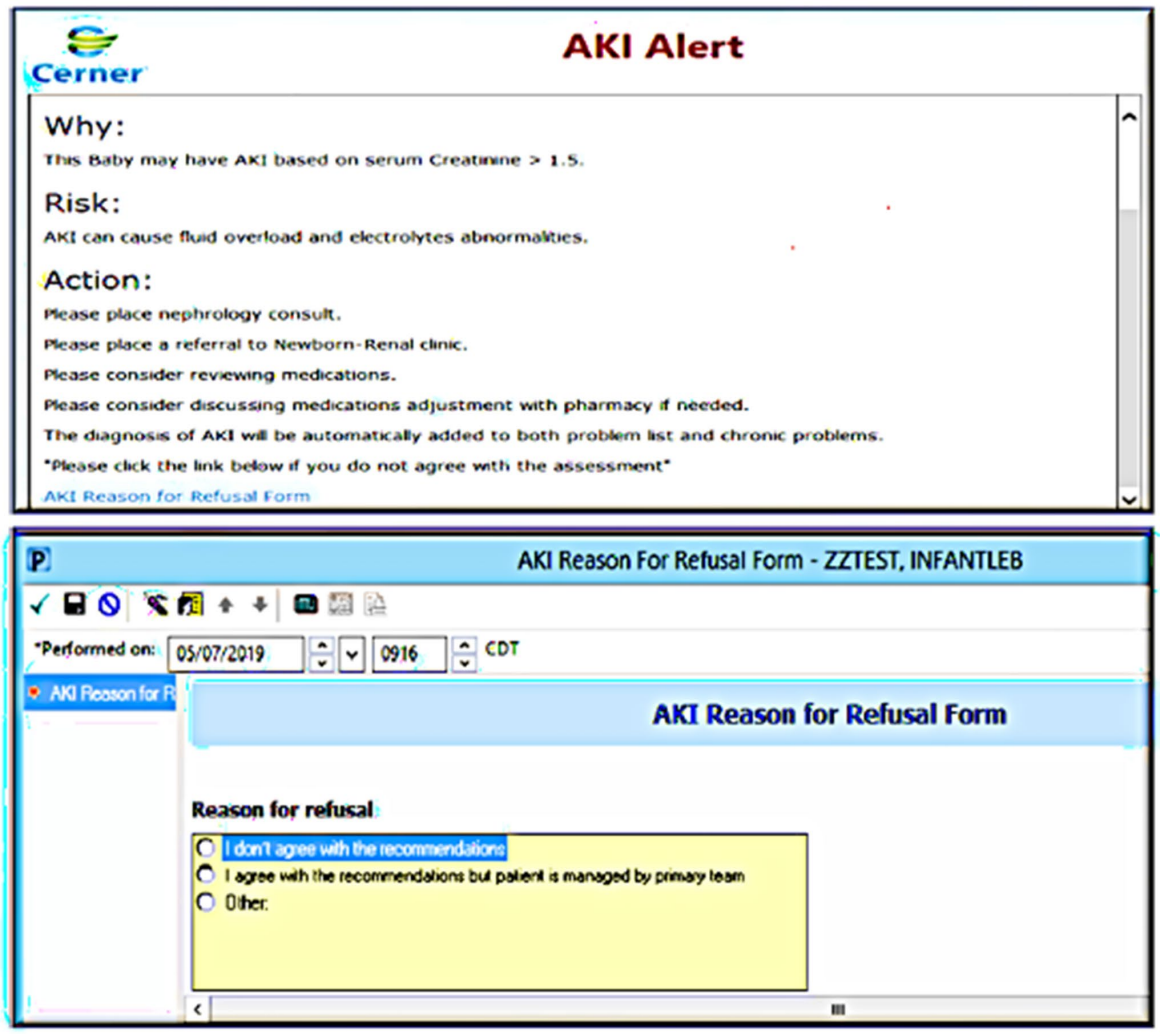
Electronic medical record acute kidney injury (EMR-AKI) alert and decline window

In this QI initiative, we implemented a real-time automated EMR alert within the EMR (Cerner, P005, Power chart) (Fig. 2) that triggers for neonates with SCr level > 1.5 mg/dL. We have adopted a stepwise approach in this project starting with using a cutoff point of SCr > 1.5 mg/dL to avoid alert fatigue and as a first step to examine the efficiency of using this neonatal AKI alert. One concern of using the KDIGO criteria was alert fatigue which is often encountered in healthcare settings, particularly with EMR-Alerts, and it refers to the desensitization that occurs when clinicians are exposed to an excessive number of alerts. This desensitization can lead to important alerts being overlooked or ignored, potentially compromising patient care. Ancker et al. [11] explored the effects of workload, work complexity, and the frequency of alerts on alert fatigue within a clinical decision support system, as data suggests that repeated exposure to alerts, especially in high-workload and complex work environments, can significantly contribute to alert fatigue, affecting the effectiveness of clinical decision support systems in healthcare which highlights the need for optimizing alert systems to balance the necessity of conveying critical information with the risk of overwhelming healthcare providers and subsequently affecting the alert or clinical decision tools' efficiency.

The alert was designed to automatically add the AKI diagnosis to the problem list and to prompt physicians to consult with nephrology, refer neonates to the neonatal nephrology clinic, and discuss with the pharmacist adjusting medications. The EMR alert that we implemented included the potential to place orders for nephrology consultations and referrals to the neonatal nephrology clinic within the same window.

Furthermore, an instantaneous Cerner inbox message was dispatched to the principal investigator (PI) to promptly inform the team of the alert activation. This was not an interventional step but rather part of monitoring the accuracy of the alert. The alert was programmed to automatically add the AKI diagnosis to the problem list and chronic problems list and to prompt the NICU team to place a nephrology consultation and referral to the neonatal nephrology clinic on the same page. The alert also prompted the team including the pharmacy to review and adjust medication for the glomerular filtration rate (GFR) as necessary (data not reported in this work). Staff had the option to decline the recommendations (Fig. 2). Within this initiative, we have incorporated other interventions, like developing a process for clinical staff to identify and notify MD of potentially eligible patients through utilizing the EMR-AKI alert.

Urine output (UOP) criteria were tested and were found to falsely trigger the alert due to inaccuracy of the UOP recording; subsequently, UOP criteria were excluded from this alert. Before activating the EMR alert, we configured the alert to operate unobtrusively in the system’s background, directing notifications exclusively to the PI inbox for data verification. Upon daily review of these alerts and corresponding patient charts, the PI observed that the alert system was erroneously triggered in cases involving neonates with recorded diaper counts or those lacking precise input/output measurements due to their stable condition and imminent discharge. Consequently, we decided to omit UOP from our criteria at this first stage.

### Assessment of AKI incidence

The baseline pre-alert assessment duration was from January 1, 2018, to June 30, 2019. The post-alert assessment duration was from July 1, 2019, to June 30, 2023. Baseline data was collected by reviewing SCr values of all neonates admitted to the NICU before and after implementing the alert. The incidence of AKI based on the SCr criteria of the modified KDIGO criteria [12] was recorded, as well as the incidence of AKI based on SCr levels > 1.5 mg/dL.

The actual KDIGO criteria were not used for this alert because of concerns of NICU team of alert fatigue. So, for the purpose of this phase one of the project, SCr of 1.5 mg/dL was used as first step to show the efficiency and do-ability of the alert. The cutoff point of SCr > 1.5 mg/dL was used as first stage to help advance the alert in the future to apply full KDIGO criteria.

Problem lists and chronic problems for all neonates with AKI (according to SCr criteria) were reviewed to determine the incidence of documentation before and after implementing the EMR-Alert for neonates with SCr > 1.5 mg/dL. Consults and referrals to nephrology were also screened, and the incidence before and after implementing the alert was reported. We compared the incidence of AKI identification/documentation before and after implementing the alert, as well as the incidence of nephrology consultations and referral to the neonatal nephrology clinic for neonates with SCr > 1.5 mg/dL.

### Statistical analysis

The chi-square test was used to compare AKI documentation, nephrology consults, and referral to the neonatal nephrology clinic in the pre- and post-EMR-Alert phases. The results were reported as relative risk (RR) and 95% confidence intervals (CIs). A *p* value of less than 0.05 was considered statistically significant. The GraphPad Prism software version 10.0.2 was used for the data analysis.

## Results

### NICU admissions and incidence of AKI

**Fig. 3:**
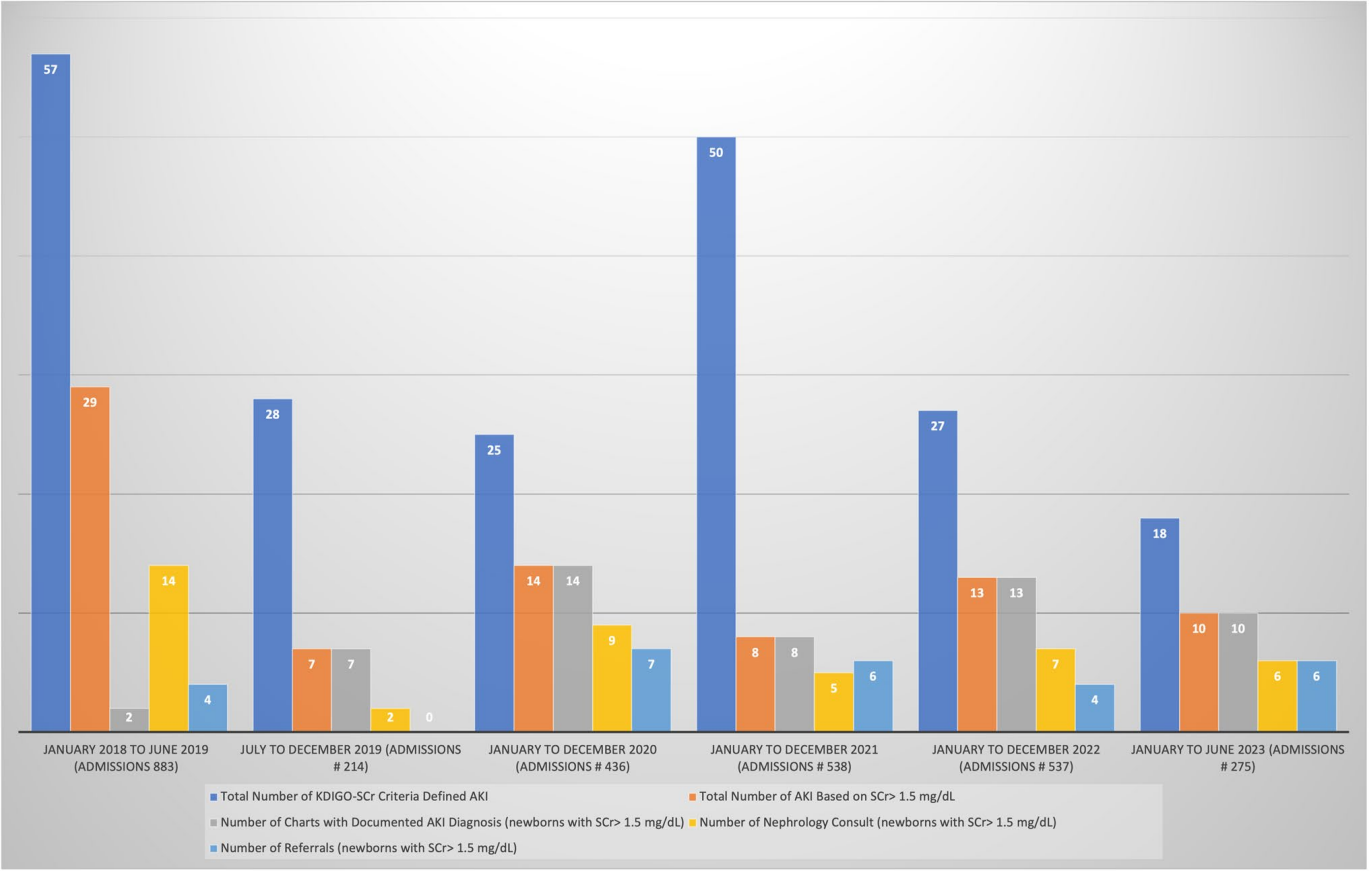
Actual numbers of yearly admissions, AKI (KDIGO serum creatinine criteria defined and serum creatinine > 1.5 mg/dL criteria), documented AKI, nephrology consults, and referrals (for newborns with serum creatinine > 1.5 mg/dL)

Figure 3 shows the yearly NICU admissions, total number of AKI based on the KDIGO-based SCr criteria, and AKI based on SCr > 1.5 mg/dL.

Before implementing the alert, 883 newborns were admitted to the NICU. The incidence of AKI based on KDIGO SCr criteria in this group was 6.5% (*n* = 57 newborns). Of the 57 newborns with AKI, 29 newborns (51%) had SCr > 1.5 mg/dL. Twelve newborns (41% of neonates with SCr > 1.5 mg /dL) died. After implementing the alert, 2000 newborns were admitted to the NICU (July 2019 to June 2023). The incidence of AKI based on the KDIGO SCr criteria in this group was 7.5% (*n* = 148). Of those with AKI based on KDIGO SCr criteria, 35% had SCr > 1.5 mg/dL. Twenty-three newborns (44% of those with SCr > 1.5 mg/dL) died. 

### Improvement in AKI documentation

**Fig. 4:**
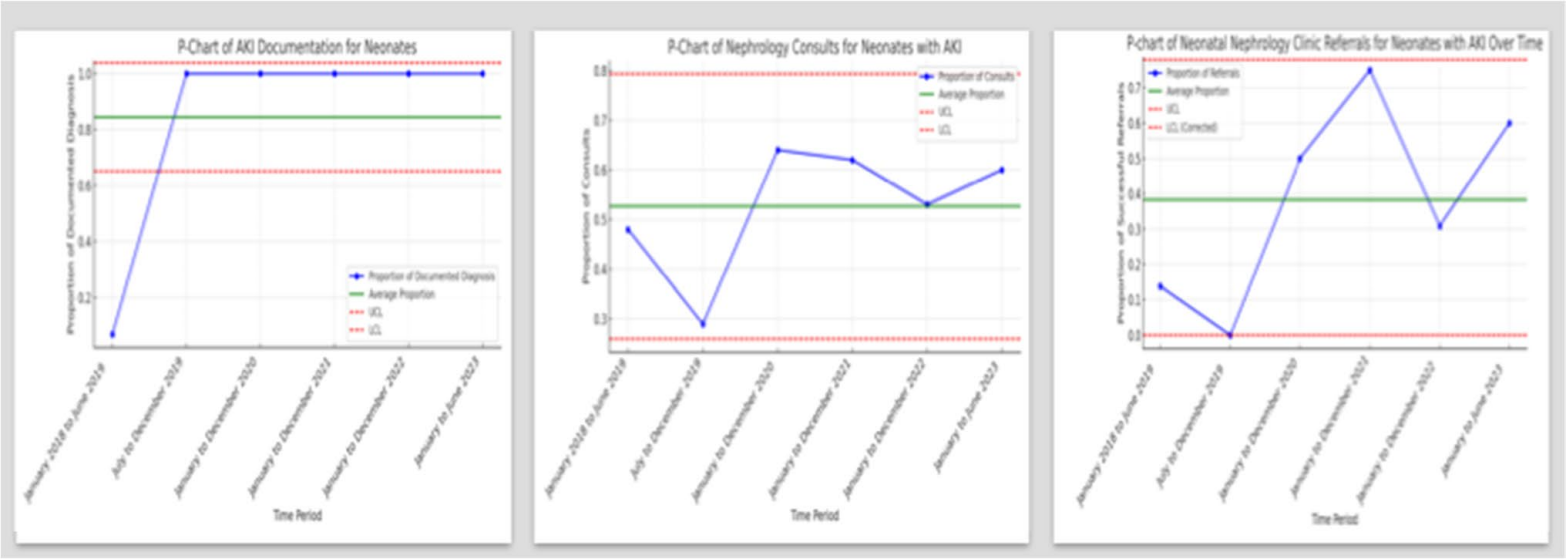
*P*-charts showing trends of percent yearly documented AKI diagnosis, nephrology consults, and referrals for newborns with serum creatinine > 1.5 mg/dL

Before implementing the alert, only 2 of 29 (7%) newborns (4% in 2018 and 20% in 2019), had an AKI diagnosis added to their problem list. This incidence significantly increased to 100% after implementing the alert, both collectively (July 2019 to June 2023) (*p* < 0.0001) and on a yearly basis, as shown in Fig. 4. Table 1 shows the yearly improvement in documentation of AKI in comparison to pre-alert time. Additionally, the alert improved the documentation for neonates with AKI based on the KDIGO SCr criteria from 14 to 56% (*p* < 0.001) which was derived mainly by the improvement of documentation in the group with SCr > 1.5 mg/dL.

**Table 1:** Comparison between yearly AKI documentation, nephrology consults, and referrals to newborn-renal clinic in comparison to the pre-alert time presented as *p* value, relative risk, and 95th confidence interval with and without correction for death

Year	Documentations ***p*** value and RR	Consults ***p*** value and RR	Consults adjusted for death ***p*** value and RR	Referrals ***p*** value and RR	Referrals adjusted for death ***p*** value and RR
2019	0.0001, + infinity 95% CI = 2 to infinity	0.3, 0.7 95% CI = 0.43 to 1.5	0.4, + infinity 95% CI = 0.4043 to + infinity	0.2, 0.86 95% CI = 0.69 to 1.2	0.5, 0.8 95% CI = 0.5274 to 3.753
2020	0.0001, + infinity 95% CI = 2 to infinity	0.3, 1.44 95% CI = 0.7 to 3.3	0.4, 1.64 95% CI = 0.5345 to 6.282	0.01, 1.7 95% CI = 1.1 to 3.2	0.05, 2 95% CI = 0.9795 to 5.728
2021	0.0001, + infinity 95% CI = 2 to infinity	0.4, 1.37 95% CI = 0.6 to 3.9	0.5, 1.647 95% CI = 0.4462 to 9.552	0.0006, 3.4 95% CI = 1.4 to 12.1	0.02, + infinity 95% CI = 1.101 to + infinity
2022	0.0001, + infinity 95% CI = 2 to infinity	0.7, 1.1 95% CI = 0.6 to 2.3	0.7, 1.23 95% CI = 0.4321 to 4.644	0.1, 1.2 95% CI = 0.9 to 2.0	0.05, 2.3 95% CI = 0.98 to 8.0
2023	0.0001, + infinity 95% CI = 2 to infinity	0.5, 1.2 95% CI = 0.6 to 3.2	0.27, 2.5 95% CI = 0.5841 to 14.48	0.003, 2.1 95% CI = 1.2 to 5.1	0.009, 4.5 95% CI = 1.269 to 25.76

### Nephrology consultations and referral to neonatal nephrology clinic among neonates with SCr > 1.5 mg/dL

Before implementing the alert, 14 newborns (48% of neonates with SCr > 1.5 mg/dL) had a nephrology consultation, but only 3 (10% of neonates with SCr > 1.5 mg/dL) were referred to the neonatal nephrology clinic. After implementing the alert, 31 newborns (47.7%) had nephrology consultations, and 25 (38.5%) were referred to the newborn renal clinic. While there was no significant increase in consultations (*p* = 0.5), there was a significant increase in referral to the neonatal nephrology clinic (*p* = 0.005). Table 1 and Fig. 4 show the yearly trends of documentation, consultations, and referrals of neonates with AKI based on SCr > 1.5 mg/dL, the *p* value, the relative risk (RR), and 95% CI compared to pre-alert baseline.

## Discussion

In this QI project, our group showed that using the EMR-AKI alert with inherited ability to add diagnosis of neonatal AKI to the problem list and chronic problem list can significantly improve the documentation of neonatal AKI (driven mainly by improvement of the documentation of AKI for neonates with SCr > 1.5 mg/dL) and referral to the neonatal nephrology clinic. The increase in consultations was not significant, which is mostly attributed to the ability of the NICU team to manage newborns with AKI who did not have other laboratory abnormalities or issues with fluid overload. Embedded within the background of the system, the alert mechanism seamlessly dispatches real-time notifications to the PI for continuous real-time monitoring. Notably, the advantage of this setup is that it operates with such an efficiency that it obviates the necessity for direct intervention by the PI. However, it is crucial to emphasize that this QI endeavor was consciously rooted in the ethos of empowering the NICU team to exercise increased autonomy. This decision was in marked contrast to the more traditional approach that might have entailed consistent intervention by nephrologists. By design, our project was conceived to foster a culture of collaboration rather than unilateral direction. The emphasis on enhancing the decision-making capabilities of the NICU team had a twofold purpose. First, it enabled the team to act promptly and judiciously in response to alerts. Second, it nurtured an environment in which the accumulation of expertise and experience within the NICU was tapped into for collective benefit. This conscious decision to avoid excessive external intervention while paradoxically invoking a more collaborative spirit internally was intended to ensure the project’s viability and relevance over time.

This project configuration recognized the ongoing success of the endeavor hinged upon embedding it within the existing workflows and decision-making structures of the NICU. Imposing a top-down approach might have yielded short-term results, but long-term feasibility could have been compromised. By cultivating a sense of ownership and collective responsibility within the NICU team, we not only facilitated smoother implementation, but also paved the way for sustained progress. In essence, our QI initiative was not merely a technical solution; it was a paradigm shift toward shared responsibility and collaboration. By empowering the NICU team to act autonomously and make informed decisions, we not only enhanced patient care in the immediate context, but also set the stage for a more resilient and adaptable healthcare approach. The deliberate choice to eschew constant external involvement in favor of internal collaboration proved to be a cornerstone of the project’s evolution, ensuring its continued relevance and impact in the dynamic landscape of neonatal care.

The use of electronic alerts linked to embedded CDS tools is one of the benefits of adopting EMR, which has been demonstrated in multiple studies: notification of critical laboratory and imaging findings, mitigation of hazardous drug interactions, enhanced use of immunizations, venous thromboembolism prophylaxis, and antiplatelet therapy among hospitalized patients, among many other utilizations [5–7]. Goldstein et al. successfully implemented an EMR screening algorithm and decision support process (trigger) among children exposed to high nephrotoxicity, resulting in a 42% reduction in AKI intensity in a single-center study and 23.8% decrease in multicenter quality improvement initiative (NINJA) [8, 13]. Similar favorable results were reported in the baby-NINJA group among neonates [9].

Our results are consistent with the published adult data. A prospective study by Holmes et al. showed that the use of an EMR-based system for patient identification overcomes the systematic underreporting of AKI associated with previous studies [14]. The QI project by Park et al., in a tertiary hospital in Korea, showed that the introduction of the EMR-Alert significantly decreased the odds of overlooked AKI events (adjusted OR, 0.40; 95%CI, 0.30–0.52), and significantly increased the odds of an early nephrology consultation (adjusted OR, 6.13; 95% CI, 4.80–7.82). Additionally, the study showed that the odds of a severe AKI event were reduced after implementation of the alerts (adjusted OR, 0.75; 95% CI, 0.64–0.89), and the likelihood of AKI recovery was improved in the alert group (adjusted HR, 1.70; 95% CI, 1.53–1.88) [15].

In a pediatric population excluding neonates, Gubb et al. showed that using AKI alerts improved the recognition of AKI [16]. Menon et al., over a 6-month pilot period, utilized an AKI alert that was sent directly to the primary provider listed for each patient (6 months to 18 years old) and showed a significant increase in AKI documentation [17].

Comprehensive documentation of neonatal AKI diagnoses serves as a pivotal link in the healthcare chain, profoundly impacting multiple facets of patient care. First, it significantly heightens the awareness of the primary care team, enabling them to swiftly identify high-risk neonates. This heightened vigilance empowers primary care physicians to take proactive measures, including essential follow-up consultations with nephrologists. These follow-up appointments are pivotal in screening for the potential development of long-term complications such as hypertension, proteinuria, and CKD.

Beyond the medical realm, meticulous documentation is vital. It acts as a conduit of information, ensuring that families are well informed about their child’s condition. This newfound awareness equips families with valuable knowledge about the nature of their children’s health challenges and the importance of continued medical surveillance. Consequently, it can significantly enhance adherence to follow-up appointments and treatment regimens, thereby promoting the overall well-being of neonates. In essence, documentation in the context of neonatal AKI is not merely a record-keeping exercise; it is a linchpin that unites healthcare providers, families, and the patient in a collaborative effort toward comprehensive care. It empowers healthcare teams to intervene proactively, shields against potential long-term complications, and ensures that families actively participate in their child’s healthcare journey. This multifaceted role of documentation underscores its indispensable significance in optimizing the health outcomes and quality of life of neonates with a history of AKI. Finally, accurate documentation of AKI diagnosis holds substantial significance, particularly within the realm of multicenter studies and databases reliant on the International Classification of Diseases (ICD) codes. This meticulous documentation not only enhances the reliability of data extracted from diverse medical settings, but also amplifies the robustness of cross-institutional analyses. In this interconnected age of healthcare research, where collaborative investigations span various healthcare facilities, the precision of AKI diagnosis documentation contributes profoundly to the integrity and comprehensiveness of the datasets underpinning critical research initiatives. By establishing a consistent and reliable foundation of diagnostic information, healthcare practitioners, researchers, and policymakers can gain more accurate insights, draw meaningful comparisons, and derive well-informed conclusions, ultimately advancing our understanding of AKI and its management on a larger scale.

To the best of our knowledge, this is the first neonatal EMR-AKI alert that is linked with automatic documentation of AKI, prompting consultations, and referral to neonatal nephrology clinics to mitigate the problem of under-documentation and loss of follow-up. To the best of our knowledge, our initiative represents a pioneering effort in the neonatal realm, an EMR alert system intricately integrated with automated documentation of AKI occurrences. This novel approach extends beyond conventional methods by seamlessly incorporating not only timely alerts, but also comprehensive mechanisms for documenting AKI diagnoses, orchestrating consults, and facilitating referrals to specialized neonatal nephrology clinics. This multifaceted innovation is meticulously designed to address a critical challenge that has long plagued the field: the issue of under-documented AKI cases and the subsequent loss of follow-up. By forging this innovative linkage between EMR-based alerts and automatic documentation, we transcend the traditional boundaries in neonatal care. This integrated system is poised to usher in a new era of streamlined and coordinated responses to AKI incidents, elevating the standard of care and bolstering the potential for positive patient outcomes. No longer will the intricacies of AKI diagnosis and the complexities of referral slip through the cracks. Instead, our comprehensive approach ensures that each step in the diagnostic and management process is seamlessly captured, ensuring a meticulous record of patient care that is invaluable for clinicians, researchers, and administrators.

This endeavor marks a significant leap forward in harnessing technology to address critical gaps in healthcare management, thereby fostering a more thorough understanding of AKI in the neonatal context and fostering improved outcomes across the spectrum of neonatal care. Through this pioneering fusion of EMR alerts and automated documentation, we set the stage for a transformative shift in neonatal AKI management, one that champions efficiency, precision, and comprehensive care delivery, ultimately forging a path toward enhanced patient well-being and future advancements in neonatal medicine. Moreover, the alert helped us identify areas of improvement, such as collaboration with neonatologists to increase early consults and the gap in diagnosing AKI using the modified KDIGO criteria.

## Conclusion

Integrating an EMR alert system with the inherent capability of appending AKI diagnoses to the problem list can offer an economical and efficient solution. This approach prompts healthcare providers to engage with nephrology teams and arrange follow-up appointments. This is a streamlined method that optimizes physician efficiency and eliminates the need for additional personnel. It is essential to underscore that the EMR alert does not aim to supplement clinical judgment; rather, it serves as a complementary tool, streamlining workflows, conserving valuable provider time, and acting as a reminder. With suitable modifications, this tool has the potential to enhance the early identification of AKI cases. Additionally, the data gathered from EMR-Alerts hold a substantial value. Beyond its immediate utility, it can be harnessed to uncover care gaps, steer the development of targeted interventions, and establish benchmarks for evaluating the efficacy of these measures. We believe that the widespread use of similar approaches can improve patient outcomes, enhance documentation accuracy, and support quality improvement initiatives.

## Limitations

The use of SCr cutoff limit of > 1.5 mg/dL was the main limiting factor of this project, However, it has increased awareness of the diagnosis of neonatal AKI, increased documentation and referral to newborn-renal clinic and paved the way to our stage II of this project, identifying AKI based on the actual modified neonatal KDIGO criteria.

## Future directions

The incorporation of the newly modified neonatal KDIGO AKI criteria into the alert structure, including the use of the UOP criteria, holds the promise of achieving a 100% improvement in documentation accuracy. The diagnostic challenge posed by the UOP criteria for neonatal AKI in NICUs has been a long-standing concern. However, by integrating a noninvasive urine collection methodology with the EMR alert system, there is a strong potential to enhance documentation accuracy. This approach not only ensures the prompt involvement of nephrology specialists, but also paves the way for improved follow-up care for neonates.

In this study, spanning from July 2019 to June 2023, we observed that adopting an EMR-AKI alert based on SCr criteria of > 1.5 mg/dL did not significantly disrupt clinical workflows. Specifically, a total of 27 neonates were referred to the newborn-renal clinic during this period, demonstrating manageable integration into existing clinical processes. Hence, had we applied the KDIGO SCr criteria, it would have resulted in a total of 148 neonates, and the follow-up requirements in the clinic would have been well accommodated. These findings underscore the practicality of incorporating the KDIGO criteria into neonatal AKI detection protocols. Importantly, this approach did not impose undue strain on either nephrology or neonatology services. This evidence strongly supports progressing to stage II of our project, where the alert system will be expanded to include all newborns meeting the KDIGO AKI criteria, reinforcing its clinical significance without overburdening healthcare providers. Adopting the PDSA approach to this project, with the initial “compromise” (using SCr of > 1.5 mg/dL) was part of our “Study” phase, where we established a starting point that is feasible and acceptable to neonatology physicians and nurse practitioners. As the project progresses through the “Do” and “Study” phases, the results and impacts of this initial criterion were evaluated as shown in this work. Based on these evaluations in the “Act” phase, we have decided to adjust the criteria to reflect KDIGO AKI criteria, reflecting a gradual, stepwise approach to improving care.

This structured iterative method allows for manageable and measured changes, ensuring that improvements are sustainable and well integrated into clinical practice. It provides a framework for justifying initial compromises as necessary steps in a larger process of continual improvement.

While the implementation of this project relies on the use of EMR, which may not be universally available worldwide, alternative innovative strategies can be explored. For instance, utilizing notes in patient charts with a designated SCr cutoff point can serve as an effective reminder for the healthcare team. To amplify the impact of this initiative, collaboration among multiple medical centers in adopting such a system can yield valuable insights into the prevalence and incidence of neonatal AKI across different patient profiles and facilitate timely intervention.

Embracing these innovative approaches can foster better interdisciplinary collaboration among healthcare teams, thereby enhancing patient care outcomes. Furthermore, it is worth noting that the comprehensive documentation of diagnoses can contribute to more accurate hospital billing. This is particularly relevant for mild stage I AKI cases that often go unnoticed, unrecorded, or unbilled.

In summary, the incorporation of modified AKI criteria, coupled with innovative methods and collaborative efforts, has the potential to revolutionize the landscape of neonatal AKI diagnosis and care. Through these advancements, healthcare professionals can strive for higher accuracy in documentation, improved patient outcomes, and more informed decision making.

### Supplementary Information

Below is the link to the electronic supplementary material.

Graphical abstract (PPTX 510 KB)

## Data Availability

The datasets generated during and/or analyzed during the current study are available from the corresponding author on reasonable request.
