# Supplementary material for: Utilizing electronic medical records alert to improve documentation of neonatal acute kidney injury
Source: Pediatr Nephrol. 2024 Mar 22;39(8):2505–14. doi: 10.1007/s00467-024-06352-2 (PMC11199246; doi:10.1007/s00467-024-06352-2)
Supplement: Supplementary file 1 — Graphical abstract (PPTX 510 KB) [file 467_2024_6352_MOESM1_ESM.pptx]

## Slide 1
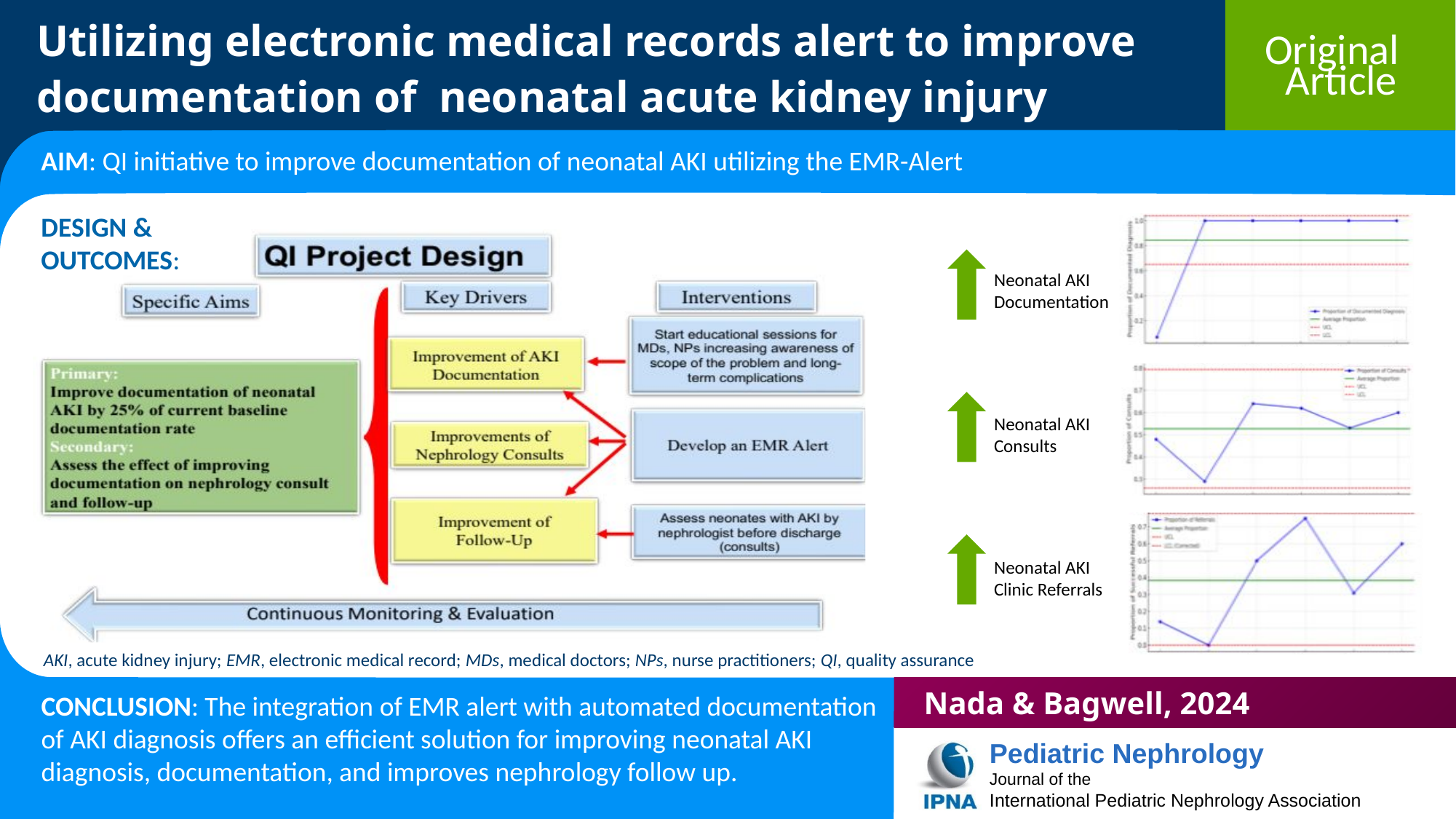

Utilizing electronic medical records alert to improve documentation of neonatal acute kidney injury
AIM: QI initiative to improve documentation of neonatal AKI utilizing the EMR-Alert
DESIGN & OUTCOMES:
Neonatal AKI Documentation
Neonatal AKI Consults
Neonatal AKI
Clinic Referrals
AKI, acute kidney injury; EMR, electronic medical record; MDs, medical doctors; NPs, nurse practitioners; QI, quality assurance
Nada & Bagwell, 2024
CONCLUSION: The integration of EMR alert with automated documentation of AKI diagnosis offers an efficient solution for improving neonatal AKI diagnosis, documentation, and improves nephrology follow up.
